# Quantities of Intra- and Extracellular DNA Reveal Information About Activity and Physiological State of Methanogenic Archaea

**DOI:** 10.3389/fmicb.2020.01894

**Published:** 2020-08-05

**Authors:** Magdalena Nagler, Sabine Marie Podmirseg, Markus Mayr, Judith Ascher-Jenull, Heribert Insam

**Affiliations:** Institute of Microbiology, Universität Innsbruck, Innsbruck, Austria

**Keywords:** extracellular DNA, intracellular DNA, microbial activity, methanogenic archaea, physiological stress

## Abstract

Although being a common aim of many microbial ecology studies, measuring individual physiological conditions of a microbial group or species within a complex consortium is still a challenge. Here, we propose a novel approach that is based on the quantification of sequentially extracted extracellular (exDNA) and intracellular DNA (iDNA) and reveals information about cell lysis and activity of methanogenic archaea within a biogas-producing microbial community. We monitored the methane production rates of differently treated batch anaerobic cultures and compared the concentrations of the alpha subunit of the methyl coenzyme M reductase gene of methanogenic archaea in extracellular and intracellular DNA fractions and in the classically extracted total DNA pool. Our results showed that this fine-tuned DNA approach coupled with the interpretation of the ratio between free exDNA and iDNA considerably improved microbial activity tracking compared to the classical extraction/quantification of total DNA. Additionally, it allowed to identify and quantify methanogenic populations that are inactive and those that are strongly influenced by cell lysis. We argue that despite the need of further studies, this method represents a novel approach to gain specific physiological information from a complex environmental sample and holds the potential to be applied to other microbes of interest.

## Introduction

Activity assays targeting the entire microbial community such as respiration measurements, live/dead staining or enzyme activity determinations are well established (Blagodatskaya and Kuzyakov, 2013), but the assessment of specific activities of microbial groups or single species within a complex community remains challenging. The most sophisticated method is the quantification of RNA that encodes for the group or microbe of interest. It is assumed that only the actively dividing members of a mixed microbial community produce rRNA (and mRNA; used to study specific enzymatic activities), and using pure cultures, the direct relationship between RNA and activity has been shown. These methods, however, come with severe limitations owed to the not always straight forward correlation between rRNA and activity in mixed cultures (summarized by Blazewicz et al., 2013). Further, the more intrinsic molecular instability and the low cellular concentrations of mRNA (Steven et al., 2017) limit routine application. Once released from intact cells, RNA is quickly degraded by RNAses, often impairing the extraction of a representative amount of RNA from environmental samples (Johnson et al., 2012). Next to relatively low RNA-yields sometimes hampering the generation of reliable results, the procedure is complex and the cost of RNA extraction is high compared to DNA extraction.

While the RNA of a specific microbe of interest correlates with activity at least in pure cultures, the amount of DNA does not directly correlate with microbial activity or growth. The total DNA pool (totDNA) is made up of intracellular DNA (iDNA) of intact cells that represent potentially active cells but include also spores and other dormant cells. Besides iDNA, every environmental sample contains extracellular DNA (exDNA) being present outside of intact cell membranes (Pietramellara et al., 2009). Still, concentrations of totDNA are widely used as a quantitative proxy in microbial ecology (Fornasier et al., 2014), but if downstream analyses are based on totDNA, the results may be severely distorted (Carini et al., 2016), since exDNA is known to be present in many habitats in relevant concentrations, sometimes even exceeding those of iDNA (Nagler et al., 2018a).

Cells release exDNA actively or passively by secretion through intact cell walls or by release after cell lysis, respectively. Actively secreted exDNA can perform a number of functions and is protected from DNAses by conformational changes or binding to the extracellular matrix (Nagler et al., 2018a). The roles of exDNA include mechanisms to repair damaged genomes, horizontal gene transfer, quorum sensing, the initial adhesion and aggregation of bacteria on surfaces during biofilm formation, the overall structural stabilization of biofilms and physical protection from pathogens (Ibáñez de Aldecoa et al., 2017; Nagler et al., 2018a). Soil exDNA may survive more than 1000 years outside of intact cells (Agnelli et al., 2007). Hence, it might prevail even if the microbe of origin has long disappeared. Furthermore, the spatial flexibility of exDNA allows movement within the habitat, e.g., dissolved in percolation water along a soil profile (Agnelli et al., 2004; Ascher et al., 2009a), with potential evolutionary implications such as genetic exchange by transformation (Pietramellara et al., 2009; Nagler et al., 2018a). It has been argued that this might have biased numerous ecological studies in terms of potential masking effects of exDNA over iDNA during downstream analyses (Fierer, 2017; Nagler et al., 2018a).

A separate extraction of exDNA and iDNA allows for a more thorough understanding and quantification of specific microorganisms’ roles in an ecosystem. Recent studies used a ratio of exDNA:iDNA as a proxy for microbial activity (Gomez-Brandon et al., 2017a,b). Nagler et al. (2018b), however, suggested the use of free DNA (fDNA), i.e., the fraction of exDNA not bound to any particles to track microbial activity, as they found, that fDNA amounts correlate best with activities, even exceeding correlations of iDNA. They argued that actively secreted exDNA is better protected from degradation than exDNA derived from cell lysis.

Little is known about active DNA secretion (extrusion) in microbiology: it was suggested that it occurs through a type IV secretion system (Hamilton et al., 2005) and is stimulated by the quorum-sensing system (Ibáñez de Aldecoa et al., 2017). Nearly all bacterial and some archaeal cells possess type IV secretion systems for DNA transfer (Christie et al., 2014) and active DNA-secretion was observed for many microorganisms including Gram-positive and Gram-negative bacteria, archaea and eukaryota (Ibáñez de Aldecoa et al., 2017). Furthermore, amounts of secreted DNA were found to correlate with activity of *Bacillus subtilis* (Garchitorena, 2017) and there is evidence that exDNA is mainly produced by active (i.e., dividing) cells, including methanogenic archaea (Nagler et al., 2018a). Therefore, we hypothesized that low amounts of exDNA relative to iDNA may hint to less active populations (i.e., with slowed cell-division) and vice versa, making exDNA, or, more specifically, the free exDNA (fDNA) a candidate to be used as a proxy for specific microbial activity.

Furthermore, iDNA might be better suited to track the present microbial community and/or activity than the exDNA-influenced totDNA.

**FIGURE 1:**
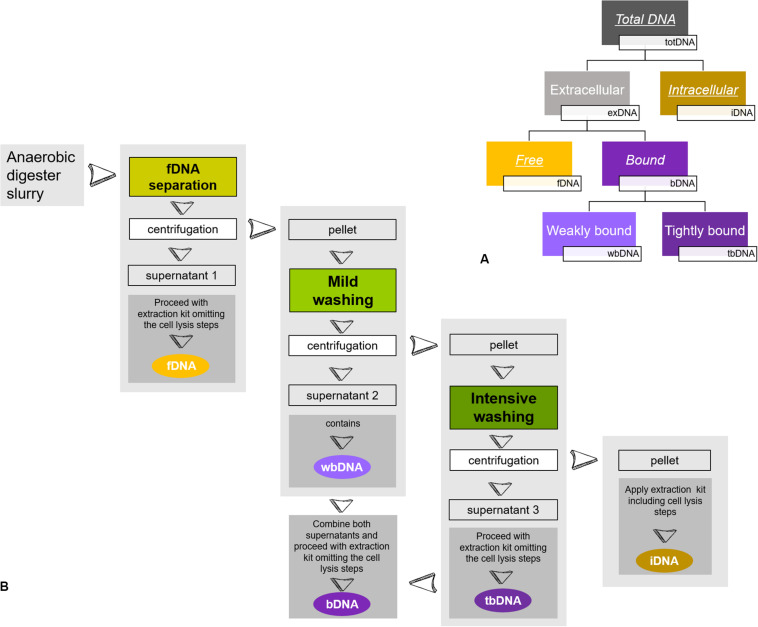
Sequential DNA extraction: **(A)** Conceptual figure of extracted DNA-fractions of the present study. While fDNA, bDNA and iDNA were extracted from the same subsample, totDNA was extracted from another subsample. Underlined DNA types were quantified via qPCR, while italics depict DNA types extracted and used for calculation of overall DNA yields. **(B)** Schematic overview of sample preparation for a sequential DNA extraction as described in Nagler et al. (2018b). During **fDNA separation**, free DNA (fDNA) that is not bound to cells or surfaces is yielded in the supernatant of the liquid digester slurry after centrifugation. Then, a **mild washing** is applied to the remaining pellet, detaching DNA weakly bound to cells or surfaces (wbDNA) using mild EDTA in PBS. A subsequent **intensive washing** of the pellet detaches DNA tightly bound to cell membrane receptors (tbDNA) by hydrolysis with trypsin and finally, intracellular DNA (iDNA) is extracted from the washed pellet. The bDNA fraction is formed by combining the supernatants of wbDNA and tbDNA. For details see Nagler et al. (2018b).

In the present study, we focused on methanogenic archaea growing within different mixed microbial communities. We monitored methane production rates as an unbiased activity parameter for methanogenic archaea on the one hand and determined methanogenic exDNA and iDNA dynamics by sequential extraction on the other hand, while comparing them to directly extracted totDNA. Further, we discriminated between exDNA bound to particles or cell walls (bDNA) and fDNA (see Figure 1A) and explored fDNA, iDNA and the fDNA:iDNA-ratio for their suitability as a proxy for methanogenic activity.

Keeping in mind that microbes undergo the classical growth phases (i.e., lag, exponential, stationary and death/lysis phase) during batch cultivation, we performed lab-scale batch anaerobic digestion experiments of three different treatments with manure and rumen fluid as inocula and assessed the parameters of interest throughout all these different microbial physiological states. By using differing C-sources and inocula within the treatments we intended to test the accuracies of the various tested fractions.

## Materials and Methods

### Experimental Setup

To obtain methanogenic communities with different activity profiles during batch cultivation, a fresh inoculum was retrieved from the biogas-plant (Gallspach, Austria) fed with a mixture of various animal manures including swine and cattle. The inoculum was allowed to outgas for 3 days and amended in three different ways. In triplicates, the inoculum was mixed with: (I) corn straw pretreated with 1.85 M NaOH (2 h, 70°C, 80% v_NaOH_/w_straw__(oDM)_) at a concentration of 16 g L^–1^ (named NaOH); (II) an energetic liquid generated during hydro-thermal carbonization of corn straw with an inoculum:liquid-ratio of 5:1 (HTC); and (III) fresh rumen liquid retrieved from a slaughterhouse with an inoculum:rumen-ratio of 1.5:1 and adding 16 g L^–1^ corn straw as an additional substrate (Rumen). Of each mixture, 300 mL were filled into 500 mL-Schott flasks, the headspaces were flushed with N_2_ and set for anaerobic digestion using the AMPTS II (Automated Methane Potential Testing System, Bioprocess Control, Lund, Sweden). Methane emissions were logged over the course of 14 days and samples for DNA extraction were taken at four time points, after 20, 67, 170, and 297 h. Three samples aliquots were retrieved simultaneously; while one sample was frozen at −20°C until totDNA extraction, the sample for sequential DNA extraction was immediately pre-treated as described in section “DNA Fractionation” and stored at −20°C until extraction. The third sample was used for dry mass determination after desiccation at 105°C over night.

### DNA Fractionation

Samples retrieved from the reactors were split and used to (i) extract total DNA (totDNA; direct extraction) and (ii) fractionate the sample and extract both, exDNA and iDNA (sequential extraction). exDNA (fDNA and bDNA) and iDNA were extracted sequentially following the protocol by Nagler et al. (2018b): a known amount of sample was centrifuged for 5 min at 5000 rcf, the generated supernatant containing **fDNA**. Subsequently, the pellet was consecutively treated first with a phosphate-buffered saline (PBS, pH 7) amended with EDTA and second with a washing solution containing trypsin to disassociate DNA weakly and tightly bound to inorganic particles or cell walls (wbDNA and tbDNA). The enzymatic reaction was stopped after 5 min by adding a trypsin inhibitor solution (see Figure 1B). After each washing step, the sample was centrifuged for 5 min at 5000 rcf and both **bDNA** fractions (i.e., wbDNA and tbDNA) were yielded in the supernatant. Subsequently, wbDNA and tbDNA were mixed to generate a single bDNA fraction. The remaining pellet containing intact cells was processed by mechanical/chemical cell lysis, as described below, yielding **iDNA**. The amounts of initial sample used for the sequential extraction as well as of the fractions containing exDNA, bDNA and iDNA were recorded in order to calculate their amounts and abundances.

### DNA Extraction

Approximately 300 mg (fresh weight, FW) of sample and 300 mg of pellet (generated as described above) were used to extract **totDNA** and **iDNA,** respectively, using the Soil DNA Purification Kit (EURx, Gdańsk, Poland) following the manufacturer’s instructions with the following minor modification: Instead of using 400 and 600 μL of the supernatant at step 6 and 9, respectively, the entire supernatant was used to yield the overall sample DNA. Cell walls were broken up using a FastPrep-24^TM^ Instrument (MP Biomedicals, Santa Ana, CA, United States) as proposed in the manual (40 s, 6.0 ms^–1^).

For **fDNA** and **bDNA** samples, 300 mg of each fractionated supernatant were used; importantly, the first bead-beating, as well as any cell-lysing buffer-related step were omitted in order to avoid a cell lysis of residual intact cells. Rather, the sample was mixed with 500 μL of the buffer present in the bead tubes and centrifuged according to the manual (step 6). All remaining purification steps of the extraction protocol were performed as described above with the changes for step 9 of the manufacturer’s manual.

The quantity of all DNA extracts was measured using a Quantus^TM^Fluorometer and the QuantiFluor^®^ Dye System for double-stranded DNA (dsDNA) (Promega, Fitchburg, MA, United States). Quality of DNA was checked on 1% (w/v) agarose gels using GelGreen^TM^ staining (Biotium Inc., Fremont, Canada).

Based on the findings of earlier studies that microbial activity might be best tracked using the fDNA fraction (Nagler et al., 2018b), bDNA was only used to calculate the sum of all DNA fractions [exDNA (fDNA + bDNA) + iDNA] in terms of DNA yield and gene copy numbers for quantitative comparison to the directly extracted totDNA, but not for downstream calculations.

### Quantitative PCR (qPCR)

qPCR of all DNA fractions (*n* = 4), time points (*n* = 4) and true parallels (*n* = 3) was conducted in technical parallels (*n* = 2), on a Rotor-Gene 6000 Real-time Thermal Cycler (QIAGEN GmbH, Hilden, Germany) using the SensiFAST SYBR^®^ Hi-ROX chemistry (Bioline, London, United Kingdom) with 2 μL of 1:10 diluted DNA-extract as template and targeting the alpha subunit of the methyl coenzyme M reductase gene (mcrA) of methanogenic archaea (mlas-mod/mcrA-rev) (Angel et al., 2012), producing a 469 bp amplicon. Catalyzing the last step of methanogenesis and being present in all methanogens, it represents a perfect target gene to measure the activity of methanogenic archaea. As a standard, DNA of a pure culture (*Methanosphaera stadtmanae*; DSMZ 3091) was PCR-amplified in five replicates with the primer pairs described above. Amplicons were purified using the GenElute^TM^ PCR Clean-Up Kit (Sigma-Aldrich, St. Louis, United States) and diluted to fit the range from 10^7^ to 10^3^ gene copies μL^–1^. All standard curves exhibited a high model fit *R*^2^ > 0.999, and all qPCR runs were conducted with 40 cycles. Amplicon quality check of qPCR via melt-curve analysis was carried out by increasing the temperature from 60 to 99°C at a rate of 0.20°C min^–1^.

### Data Analysis

Methane production rates were normalized on basis of the fresh weight (FW) of the treatment-related mixtures, calculated on an hourly basis and expressed as volume at base conditions (Normal mL, NmL).

qPCR results were reported as mcrA gene copies gFW^–1^ considering the respective mass of each DNA fraction and their relative contribution to the overall sample. Amounts of the various fractions over all time points were compared in order to evaluate the extraction efficiencies on the level of total DNA yield and of mcr*A* gene copies.

To assess and correlate the dynamics of fDNA with the physiological state of the investigated methanogenic community, numbers of fDNA mcrA gene copies were investigated with regard to iDNA mcrA gene copies, calculating the fDNA:iDNA-ratio.

To our knowledge, ranges of fDNA:iDNA in different growth phases and especially of dividing and cell lysis influenced cultures have not yet been investigated. In order to take the first step, we formed three theoretical ranges based on earlier findings (Nagler et al., 2018b), to be tested within the present and following studies. First, we assumed that in exponentially growing populations of methanogens more DNA should be located inside than outside the cells and second that actively growing or proliferating cells secrete at least 1/10 of their DNA (iDNA) to perform extracellular tasks. Based on these assumptions, we applied the ratio and defined the following ranges:

• **fDNA:iDNA-ratio **>** 1 (H)**: methanogens are lysed to a high degree (i.e., fDNA is highly abundant compared to iDNA);
• **fDNA:iDNA-ratio 0.1–1 (M):** methanogens are encountering normal growth conditions (i.e., amounts of fDNA and iDNA are balanced);
• **fDNA:iDNA-ratio **<** 0.1 (L):** methanogens are inactive and may be considered as dormant (i.e., mcrA gene copies within fDNA are at least 10 times less abundant than in iDNA).

The accuracy, reliability and informative value of these ranges were tested by comparing them to the methanogenic activities as determined by their methane production rates.

To check for the accuracy of the various DNA fractions in tracking methanogenic activity, Pearson correlation coefficients (*r*) were calculated in R (R Core Team, 2018), applying a significance level of α = 0.01, for all DNA fractions and ratios. Additionally, significantly correlating DNA fractions were further investigated according to Bland and Altman (Bland and Altman, 1986) on data normalized to a range between 0 and 1.

Given a normal distribution of the data, differences between groups of interest were checked applying a one-way ANOVA and a Tukey’s pairwise *post hoc* test. If normality failed, a Kruskall-Wallis test combined with Mann-Whitney pairwise comparisons was applied.

## Results

### Methane Production Rates

**FIGURE 2:**
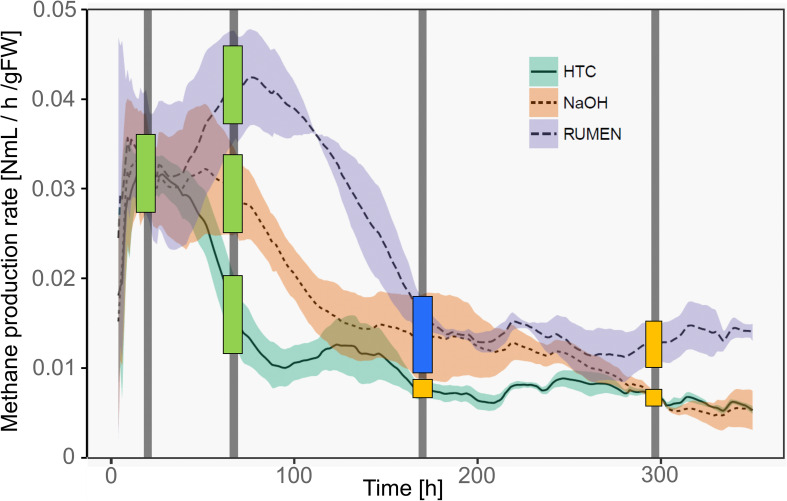
Methane production rates of the three treatments over time. Lines represent mean values, shaded areas the standard deviation (*n* = 3). Vertical bars mark the time points of sample collection (T1–T4). Colored squares depict the physiological state of the samples taken at these time points. Green, HPP (high productive phase); blue, CL (cell lysis); yellow, LPP (low productive phase).

Methane production rates were different among the three treatments. Representing different physiological states of the methanogenic archaea, samples were classified as deriving from the *high productive phase (HPP)* or the *low productive phase (LPP)*, i.e., producing >1 and <0.5% of the by then accumulated methane volume per hour, respectively. Generally, treatment HTC exhibited the overall lowest methane yields. It showed a HPP at T1 and a subsequent decrease in methane formation, while both NaOH and Rumen showed HPP spanning over T1 and T2. During T3 and T4, samples of all treatments belonged to the LPP, with NaOH and HTC decreasing until the end of the experiment and Rumen showing a stable activity throughout T3–T4 (Figure 2).

With the shift from HPP to LPP, methanogenic activities decreased and this is attributed to a decrease in the abundance and/or activity of methanogenic communities. During such a decrease, methanogens may lyse, changing the exDNA vs. iDNA characteristics of subsequent sampling events. Therefore, we introduced an additional type to characterize the physiological state, namely lysing populations *(cell lysis, CL).* This type was found in T3 samples of NaOH and Rumen treatments; in fact, sampling was performed directly after the methane production drop and thus, during the phase of highest CL and in the transition phase to LPP. For HTC, highest CL occurred around 100 h after the start of the experiment. T2 (after 67 h), as well as T3 (after 170 h), might both be influenced by CL but also show some characteristics of HPP and LPP. As a compromise facilitating the data presentation and according to their percentage of accumulated methane volume per hour, HTC T2 samples were classified as HPP and HTC T3 samples as LPP (Figure 2).

### DNA Fractions

Generally, melt-curve analysis of the amplicons did not show any indications for primer-dimers or non-specific amplifications (data not shown).

**FIGURE 3:**
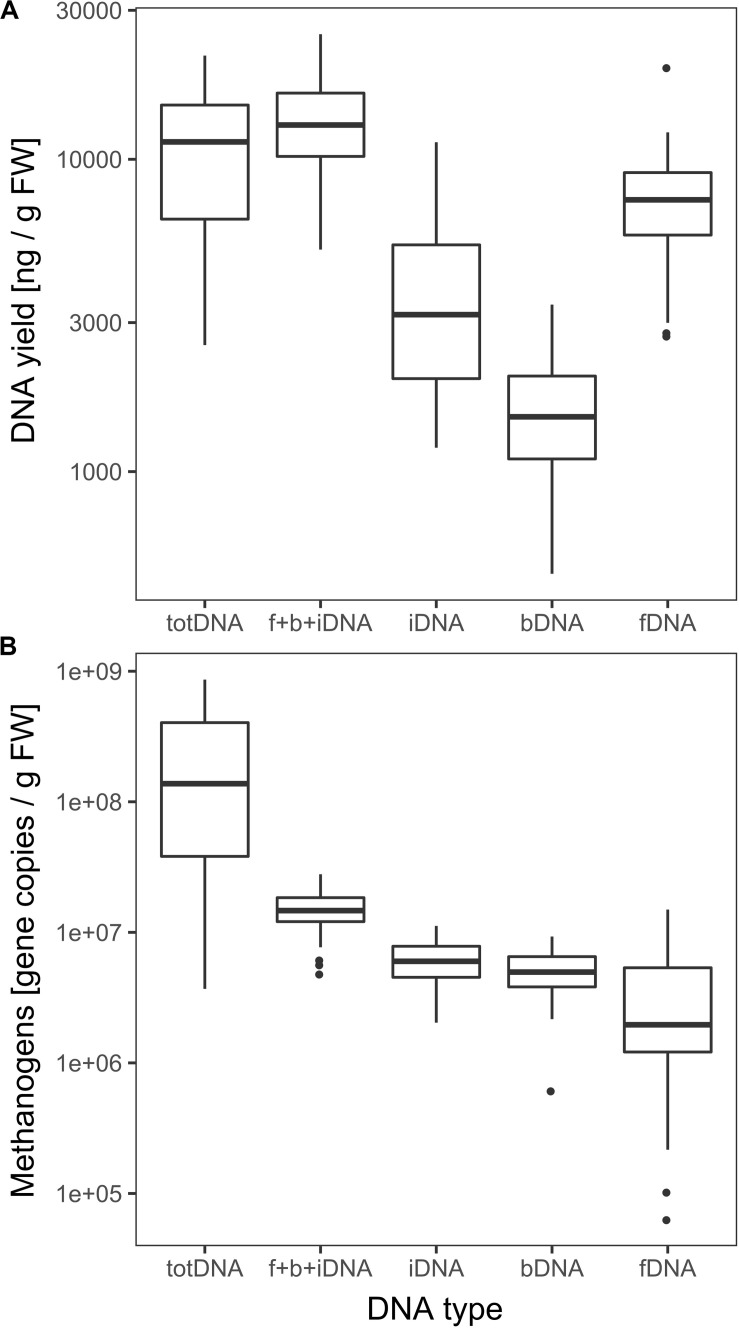
**(A)** Logarithmic overall DNA yields and **(B)** mcrA gene copies of the various DNA pools. Vertical lines represent the median, boxes the quartiles and dots the outliers of all data collected over time.

fDNA was the largest of all sequentially extracted DNA fractions with 7628 ± 3205 ng DNA gFW^–1^, followed by iDNA (4017 ± 2594 ng gFW ^–1^) and bDNA (1605 ± 647 ng gFW ^–1^) (Figure 3A). Sequential extraction (f + b + iDNA = 13,251 ± 4720 ng gFW^–1^) and classical direct extraction (totDNA; 11,408 ± 5442 ng gFW^–1^) showed similar DNA yields.

While CL samples contained similar amounts of iDNA as LPP samples, their exDNA concentration was significantly higher, exceeding those of LPP as well as HPP and resulting in a clearly increased iDNA:fDNA ratio (Table 1). These samples were characterized by a preceding steep decrease in methanogenic activity, with LPP samples showing on average 2.6 times less methane production than HPP samples. Looking at the mcrA gene abundance, this gas production drop was accompanied by a loss of mcrA gene copies in totDNA (6.3 times), f + b + iDNA (1.6 times), iDNA (1.5 times) and fDNA (2.8 times) in LPP as compared to HPP samples. For CL-samples, however, mcrA gene copies were 2.3 times increased in exDNA and 4 times in fDNA compared to non-CL samples (Table 1).

**TABLE 1:** Means and standard deviations of DNA pools (gene copies gFW-1) and the fDNA:iDNA-ratio of samples classified as deriving from the high productive phase (HPP), low productive phase (LPP) or being influenced by cell lysis (CL).

		**Overall**			**HTC**			**NaOH**			**Rumen**		
		**CL**	**HPP**	**LPP**	**CL**	**HPP**	**LPP**	**CL**	**HPP**	**LPP**	**CL**	**HPP**	**LPP**
*n*		6	18	12	0	6	6	3	6	3	3	6	3
totDNA	Mean	6.2E + 7	4.4E + 8	7.0E + 7	–	3.3E + 8	1.4E + 7	8.8E + 7	6.5E + 8	2.0E + 8	3.7E + 7	3.5E + 8	4.9E + 7
	*SD*	4.0E + 7	2.6E + 8	9.8E + 7	–	1.4E + 8	7.1E + 6	4.2E + 7	2.9E + 8	1.2E + 8	1.2E + 7	1.9E + 8	1.2E + 7
f + b + iDNA	Mean	2.1E + 7	1.6E + 7	1.0E + 7	–	1.5E + 7	8.9E + 6	2.4E + 7	1.5E + 7	8.2E + 6	1.9E + 7	1.8E + 7	1.4E + 7
	*SD*	3.6E + 6	3.6E + 6	3.9E + 6	–	2.1E + 6	3.3E + 6	3.3E + 6	2.1E + 6	2.5E + 6	1.9E + 6	4.7E + 6	2.7E + 6
fDNA	Mean	9.1E + 6	3.1E + 6	1.1E + 6	–	2.5E + 6	1.3E + 6	9.8E + 6	2.1E + 6	2.5E + 5	8.3E + 6	4.6E + 6	1.5E + 6
	*SD*	2.9E + 6	2.1E + 6	1.4E + 6	–	1.3E + 6	1.8E + 6	3.7E + 6	8.5E + 5	1.5E + 5	1.4E + 6	2.7E + 6	2.5E + 5
exDNA	Mean	1.6E + 7	8.5E + 6	5.1E + 6	–	8.4E + 6	4.6E + 6	1.8E + 7	7.9E + 6	3.2E + 6	1.5E + 7	9.0E + 6	8.0E + 6
	*SD*	3.0E + 6	2.7E + 6	2.8E + 6	–	1.4E + 6	2.7E + 6	3.1E + 6	2.1E + 6	1.5E + 5	1.5E + 6	3.7E + 6	2.0E + 6
iDNA	Mean	4.7E + 6	7.3E + 6	4.9E + 6	–	5.7E + 6	4.3E + 6	5.3E + 6	7.1E + 6	5.0E + 6	4.2E + 6	9.0E + 6	6.1E + 6
	*SD*	1.3E + 6	2.6E + 6	1.8E + 6	–	3.5E + 6	1.2E + 6	4.0E + 5	8.9E + 5	2.3E + 6	1.5E + 6	1.3E + 6	1.7E + 6
fDNA:iDNA	Mean	2.06	0.45	0.22	–	0.58	0.26	1.84	0.31	0.04	2.27	0.49	0.26
	*SD*	0.84	0.30	0.25	–	0.40	0.31	0.66	0.13	0.02	0.94	0.27	0.08

*totDNA, total DNA; f + b + iDNA, sum of fDNA + bDNA + iDNA; bDNA, bound extracellular DNA; fDNA, free extracellular DNA; exDNA, extracellular DNA; iDNA, intracellular DNA.*

DNA fragment size (molecular weight) was highest for totDNA samples, followed by iDNA, bDNA and fDNA (Supplementary Figure S1). Generally, totDNA yielded one size fraction, while sequentially extracted DNA species exhibited one band > 1 kbp (as for totDNA) and a second, smaller size-fraction. Compared to totDNA, iDNA showed higher numbers of short DNA fragments, increasing in size during T3 and T4. The degree of fragmentation (visible as smear on the agarose gel) also influenced the amount of mcrA gene copies detected via qPCR: while totDNA contained about 16 times more mcrA gene copies (2.6 × 10^8^ ± 2.7 × 10^8^ gene copies gFW^–1^, Figure 3B) than f + b + iDNA (1.5 × 10^7^ ± 5.3 × 10^6^ gene copies gFW^–1^), this proportion was depending on the treatment and on the physiological state of the community (Supplementary Figure S2). While HPP samples showed the highest discrepancies between both proportions (between 20.4 and 49.4), f + b + iDNA amounts of CL samples were almost equal to totDNA samples (1.7–3.6). Furthermore, NaOH samples showed highest discrepancies, followed by HTC and Rumen (Supplementary Figure S2).

### Relationships of DNA Pools and Methanogenic Activities

**FIGURE 4:**
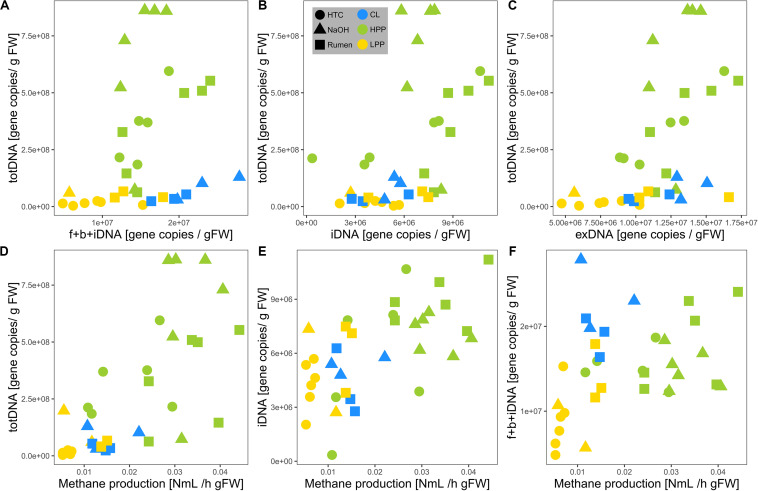
Relationships of mcrA gene copy numbers of **(A)** f + b + iDNA, **(B)** iDNA, and **(C)** exDNA with totDNA and of mcrA gene copy numbers of **(D)** totDNA, **(E)** iDNA, and **(F)** f + b + iDNA with measured methane production rates. Colors are based on the methanogenic activity levels (HPP, high productive phase; LPP, low productive phase; CL, cell lysis-influenced samples) while shapes correspond to treatments.

TotDNA and f + b + iDNA were not linearly correlated (Figure 4A): while LPP and CL samples showed a flat linear relationship, HPP samples were inflated, forming a second, steeper relationship. In detail, iDNA showed a linear relationship with totDNA (Figure 4B), while exDNA reflected the pattern of f + b + iDNA with two distinct relationships (Figure 4C). exDNA and iDNA from HPP and LPP samples showed a positive linear correlation, while CL samples tended to show increased exDNA levels with regard to iDNA (Supplementary Figure S3A).

**FIGURE 5:**
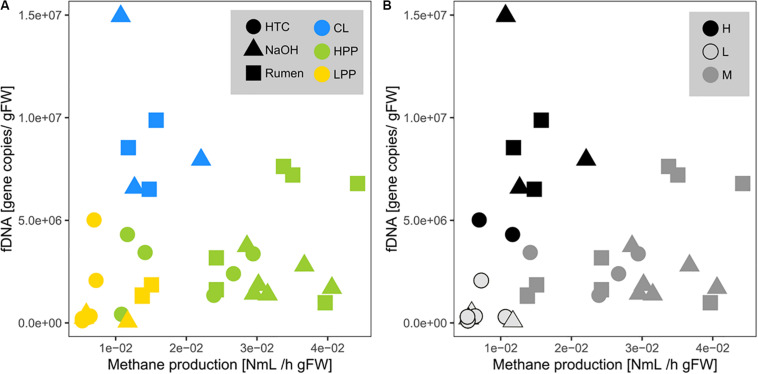
Relationship of mcrA gene copies within fDNA with methane production rates (= activity of archaeal methanogens). Point colors are based on **(A)** the classifications done by methanogenic activity profiles (HPP, high productive phase; LPP, low productive phase; CL, cell lysis-influenced samples) and on **(B)** the fDNA:iDNA-ratio of respective samples (*H* > 1, *M* = 0.1–1, *L* < 0.1). Shapes correspond to treatments.

Methane production showed exponential and linear correlations with totDNA (*p* < 0.05, *R*^2^ = 0.44) (Figure 4D) and iDNA (*p* < 0.05, *R*^2^ = 0.50) (Figure 4E), respectively. No correlation was found with f + b + iDNA (Figure 4F), however, when CL samples were excluded, a linear relation became evident. Similarly, methane production revealed no prominent correlation with fDNA (Figures 5A,B). Independent of their classification into HPP, LPP or CL, samples with low methane production rates and high fDNA concentrations were found to exhibit a fDNA:iDNA-ratio > 1 (Figure 5B, “H”), while samples with low methane production and low fDNA amounts were found to exhibit fDNA:iDNA-ratio < 0.1 (Figure 5B “L”). In addition to all samples classified as CL, two of the HTC samples of T2 and T3 showed a fDNA:iDNA-ratio > 1. fDNA:iDNA was further found to be statistically different among all physiological states (i.e., HPP, LPP, and CL, Supplementary Figure S3B).

In total, nine samples out of 36 were found to exhibit a fDNA:iDNA-ratio > 1 (i.e., HTC1_T1, HTC3_T2 and HTC2_T3, all NaOH_T3 and all Rumen_T3 samples), ranging between 1.02 (HTC2_T3) and 3.56 (Rumen2_T3). These stress-related peaks in fDNA:iDNA were observed during the stage of methane production drop (Supplementary Figure S4, HTC, Rumen) or shortly after (Supplementary Figure S4, NaOH).

A fDNA:iDNA-ratio < 0. 1 was found for seven samples (HTC3_T3, all HTC_T4- and all NaOH_T4- samples) and ranged between 0.079 (HTC1_T4) and 0.023 (NaOH3_T4).

To further test the hypothesis that fDNA increases with microbial activity if not deriving from cell lysis, fDNA amounts of the samples influenced by cell-lysis were recalculated multiplying respective iDNA amounts by the mean fDNA:iDNA-ratio of non-cell-lysis-influenced samples of the same treatment and generating a new fDNA (fDNAnew).

**FIGURE 6:**
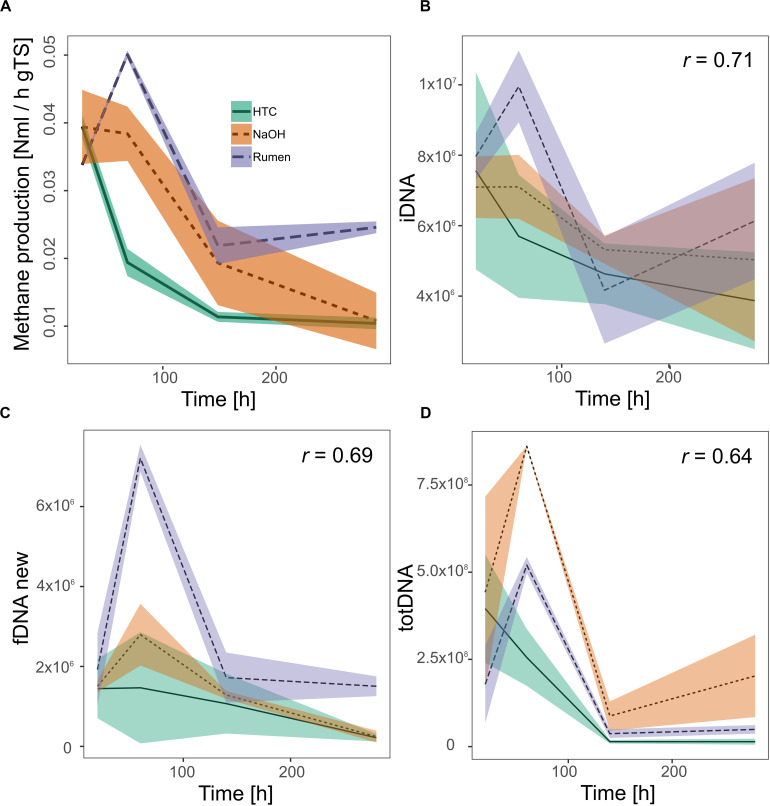
Activity of methanogenic archaea as measured by their methane production rates **(A)** and as approximated quantifying mcrA gene copies (gene copies gFW^– 1^) within **(B)** iDNA, **(C)** fDNAnew, and **(D)** totDNA. All significant Pearson correlation coefficients (r, *p* < 0.01) of the approximations with the methane production rates are given in the respective graphs. Lines represent mean values, shaded areas the standard deviation (*n* = 3).

Investigating all DNA-pools and -ratios, significant correlations with methane production were found for iDNA (*r* = 0.71), fDNAnew (*r* = 0.69), and totDNA (*r* = 0.64) (Figure 6). Generally, all models tended to overestimate low activities and to underestimate high activities. While iDNA exceeded the ± 2 SD threshold as proposed by Bland and Altman (Bland and Altman, 1986) only occasionally, fDNAnew and totDNA showed some significant deviations from methane production, especially in case of T1 samples of various treatments, showing lower relative activities than suggested by the results of methane production (Supplementary Figure S5).

## Discussion

The time- and treatment related methane production rates led to characteristic activity profiles of the methanogenic archaea, thus allowing to investigate fDNA and iDNA dynamics and associated information on the microbial activity. For the interpretation of the complex results it is important to discriminate between the physiological state of the methanogenic communities, i.e., samples that exhibit high (HPP) or low methanogenic activity (LPP) and samples that are influenced by cell lysis (CL), characterized by a preceding steep decrease in methanogenic activities.

While DNA yields were comparable between the classical extraction approach (direct extraction; totDNA pool) and the sum of sequentially extracted DNA fractions, intracellular DNA quality in terms of fragment size was higher for totDNA, matching the observation of a previous study (Nagler et al., 2018b). Methodologically, from the pellet remaining after the “elimination/recovery” of exDNA, iDNA was extracted from the cells after three centrifugation steps, a mild trypsin and EDTA treatment followed by an intensive bead beating (see Figure 1). totDNA, however, was extracted by direct bead-beating (i.e., without any pre-treatment). Keeping in mind that other studies yielded increased cumulative DNA amounts from sequential extraction as compared to the classical one (Wagner et al., 2008; Ascher et al., 2009b; Nagler et al., 2018b), these results hint toward an increased susceptibility of sequentially extracted cells to cell lysis and hence to DNA-fragmentation induced by bead-beating. Therefore, it is advised to reduce the intensity (speed and/or time) of the bead-beating or to apply a gentler method to break up cell walls.

Due to reduced fragment sizes, qPCR results differed considerably between both extraction approaches, where totDNA contained greater amounts of intact DNA sequences resulting in more mcrA gene copies than f + b + iDNA together. Compared to f + b + iDNA, totDNA was highly abundant in HPP samples, but less abundant in CL samples in terms of target gene sequences amplifiable via qPCR (c.f. Figure 4A). This might be due to the classical extraction procedure that damages part of the exDNA during the first extraction steps, when lysis buffers and bead beating disrupt cells. Classical extraction degrades exDNA during these first steps which are skipped in sequential extraction, thus resulting in higher amounts of cumulative f + b + iDNA. With exponential growth and large amounts of intact cells and thus iDNA in HPP samples, totDNA contained far more qPCR-amplifiable DNA fragments than the fractionated samples, while LPP samples laid in between. Accordingly, totDNA and f + b + iDNA comparisons do not follow a linear increase, but show two separate patterns, i.e., CL samples featuring a shallow slope, HPP samples a steep slope and intermediate LPP samples being connected with both, CL and HPP samples (c.f. Figure 4A).

At first, methanogenic communities were exponentially proliferating and building up large amounts of iDNA but also fDNA during HPP. Then, the exponential growth was stopped due to the batch fermentation approach and hence limited nutrient availability. Next to possibly being incorporated by predatory or scavenger bacteria, excessive methanogenic cells were to a large extend undergoing cell lysis, as supported by increased exDNA- and decreased iDNA concentrations in CL- with regard to HPP samples. Finally, methanogens in LPP samples were growing slowly, in a community with decreased density and releasing lower amounts of exDNA.

As mentioned above, mcrA gene copy numbers were higher in totDNA as compared to f + b + iDNA, especially for HPP NaOH samples (Figure 4A). Accordingly, totDNA as an activity measurement overestimates NaOH activity with regard to Rumen (Figure 6E).

Due to the dynamic formation of exDNA, the intracellular DNA fraction (iDNA) is suggested to track the methanogenic activity more specifically than totDNA (containing both exDNA and iDNA). In fact, relationships of methanogenic activity with totDNA exhibited an exponential character, while iDNA and f + b + iDNA (except CL-samples) both showed a linear one. totDNA is thereby greatly influenced by high amounts of mcrA gene copies in the iDNA pool of HPP samples (Figure 4B) and only to a minor extent by changing levels of exDNA, as mcrA gene copies in the exDNA pool of HPP samples were not particularly high as compared to totDNA (Figure 4C). iDNA is adjusted for that bias; in fact, they show a clear linear relationship with methanogenic activity.

The interpretation of the observed relation between fDNA and methanogenic activity is even more complex. Although it was suggested in a previous study that the amounts of fDNA provide a proxy for microbial activity (Nagler et al., 2018b), the present study reveals that the relationships of fDNA with methanogenic activity are strongly influenced by CL samples (Figure 5). In fact, in our batch fermentation approach, all treatments induced nutrient exhaustion that subsequently caused a steep decline of the methanogenic population, accompanied by an increase in fDNA. These samples experiencing a high stress might mask the relationship of fDNA with methanogenic activity that was found to be balanced under normal growth conditions.

Indeed, another evidence for the fDNA:iDNA*-*ratio as a sensitive descriptor of the physiological state of microbial cells is the fact that samples with a fDNA:iDNA-ratio > 1 matched the samples that were initially classified as CL samples and moreover allowed to identify two samples from HTC treatment, where an initial categorization was impossible.

The fDNA:iDNA*-*ratio was further confirmed by focusing on samples with a fDNA:iDNA-ratio < 0.1, which was found to be a reliable indicator of microbial inactivity. These mostly inactive or dormant methanogenic populations were characterized by the predominance of intact cells (high concentrations of iDNA) with low potential to extrude DNA (low concentration of exDNA) and were represented by samples producing not more than 0.2% of the total accumulated methane volume per hour or 0.008 NmL CH_4_ h^–1^ g^–1^ DW.

The in-depth evaluation of the correlations with methanogenic activity further confirmed that the classical molecular approach based on totDNA draws a biased picture of methanogenic activity. As discussed above, not only the relative amounts between the treatments were inverted (i.e., NaOH seemed to be more active than Rumen) but also the heights of the initial peaks and the trends toward the end of the experiment were misleading; while NaOH seemed to gain activities during T4, Rumen and HTC were both inactive through T3 and T4. In comparison to totDNA, iDNA better reflected activities toward the end of the experiment (T4) as well as relative differences among treatments.

Calculating the fDNA:iDNA-ratio we were able to specifically detect CL-biased results (i.e., with a fDNA:iDNA-ratio > 1) and to re-calculate their theoretical fDNA concentrations without cell lysis. This procedure mainly served as a test for the hypothesis that fDNA amounts are correlated to activity. Indeed, it allowed tracking microbial activities using fDNAnew although cell lysis was occurring. Activity tracking showed higher correlation values compared to totDNA, but exhibited major deviations from methane production rates during T1, when activities were highly underestimated.

Summing up, this experiment showed that a comparative, discriminatory investigation of iDNA and fDNA offers details on the physiological state of the investigated microbial group that would be masked if only totDNA were analyzed. Within the present study, a fDNA:iDNA-ratio > 1 indicated cell-lysis-influenced or stressed communities, while a fDNA:iDNA-ratio < 0.01 pointed toward inactive (incl. dormant) methanogenic populations. The proposed thresholds are preliminary, however, as they were deduced by the investigation of only a single microbial group within three representative habitats. Following further investigations, we believe, however, that our approach will be applicable to other microbial groups in various habitats.

Generally, the present data support the hypothesis that our fine-tuned DNA extraction approach, the separate analysis of the sequentially extracted extra- and intracellular fractions of the total DNA pool coupled with the presented models allows to delineate the physiological state of a specific group within a complex community.

Specifically, (i) iDNA is better suited to track methanogenic activity than totDNA; (ii) free exDNA (fDNA) is correlated with microbial activity as long as the community is not subjected to cell lysis; and (iii) calculating fDNA:iDNA provides physiological details that are missed by solely studying the directly extracted totDNA.

We conclude that fDNA:iDNA-ratios provide a sensitive and powerful tool to monitor the process of methane production within an anaerobic digester, with the potential to routinely monitor the physiological state of the methanogenic consortium and that despite the need of further studies, this method might represent a novel approach to gain specific physiological information also from other habitats.

## Data Availability Statement

All datasets presented in this study are included in the article/Supplementary Material.

## Author Contributions

MN designed the study, performed laboratory work, evaluated and interpreted the results, and wrote the manuscript. SP helped with study design and contributed to the manuscript. MM performed essential lab work. JA-J and HI both interpreted the results and contributed to the writing of the manuscript. All authors contributed to the article and approved the submitted version.

## Conflict of Interest

The authors declare that the research was conducted in the absence of any commercial or financial relationships that could be construed as a potential conflict of interest.

## Abbreviations

bDNA: bound extracellular DNA
CL: cell lysis
exDNA: extracellular DNA
fDNA: free extracellular DNA
f + b + iDNA: summed-up extracellular and intracellular DNA fractions
HPP: high productive phase (physiological state)
HTC: hydrothermal carbonization (sample treatment)
iDNA: intracellular DNA
LPP: low productive phase (physiological state)
NaOH: Sodium hydroxide (sample treatment)
totDNA: total directly extracted DNA.

## References

[B1] Agnelli A. Ascher J. Corti G. Ceccherini M. T. Nannipieri P. Pietramellara G. (2004). Distribution of microbial communities in a forest soil profile investigated by microbial biomass, soil respiration and DGGE of total and extracellular DNA. *Soil Biol. Biochem.* 36 859–868. 10.1016/j.soilbio.2004.02.004

[B2] Agnelli A. Ascher J. Corti G. Ceccherini M. T. Pietramellara G. Nannipieri P. (2007). Purification and isotopic signatures (δ 13C, δ 15N, Δ14C) of soil extracellular DNA. *Biol. Fertil. Soils* 44 353–361. 10.1007/s00374-007-0213-y

[B3] Angel R. Claus P. Conrad R. (2012). Methanogenic archaea are globally ubiquitous in aerated soils and become active under wet anoxic conditions. *ISME J.* 6 847–862. 10.1038/ismej.2011.141 22071343 PMC3309352

[B4] Ascher J. Ceccherini M. T. Guerri G. Nannipieri P. Pietramellara G. (2009a). “e-motion” Of Extracellular DNA (e-DNA) in soil. *Fresenius Environ. Bull.* 18 1764–1767.

[B5] Ascher J. Ceccherini M. T. Pantani O. L. Agnelli A. Borgogni F. Guerri G. (2009b). Sequential extraction and genetic fingerprinting of a forest soil metagenome. *Appl. Soil Ecol.* 42 176–181. 10.1016/j.apsoil.2009.03.005

[B6] Blagodatskaya E. Kuzyakov Y. (2013). Active microorganisms in soil: critical review of estimation criteria and approaches. *Soil Biol. Biochem.* 67 192–211. 10.1016/j.soilbio.2013.08.024

[B7] Bland J. M. Altman D. G. (1986). Statistical methods for assessing agreement between two methods of clinical measurement. *Lancet* 1 307–310. 10.1016/s0140-6736(86)90837-8 2868172

[B8] Blazewicz S. J. Barnard R. L. Daly R. A. Firestone M. K. (2013). Evaluating rRNA as an indicator of microbial activity in environmental communities: limitations and uses. *ISME J.* 7 2061–2068. 10.1038/ismej.2013.102 23823491 PMC3806256

[B9] Carini P. Marsden P. J. Leff J. W. Morgan E. E. Strickland M. S. Fierer N. (2016). Relic DNA is abundant in soil and obscures estimates of soil microbial diversity. *Nat. Microbiol.* 2:16242. 10.1038/nmicrobiol.2016.242 27991881

[B10] Christie P. J. Whitaker N. González-Rivera C. (2014). Mechanism and structure of the bacterial type IV secretion systems. *Biochim. Biophys. Acta Mol. Cell Res.* 1843 1578–1591. 10.1016/j.bbamcr.2013.12.019 PMC4061277 24389247

[B11] Fierer N. (2017). Embracing the unknown: disentangling the complexities of the soil microbiome. *Nat. Rev. Microbiol.* 15:579. 10.1038/nrmicro.2017.87 28824177

[B12] Fornasier F. Ascher J. Ceccherini M. T. Tomat E. Pietramellara G. (2014). A simplified rapid, low-cost and versatile DNA-based assessment of soil microbial biomass. *Ecol. Indic.* 45 75–82. 10.1016/j.ecolind.2014.03.028

[B13] Garchitorena M. G. (2017). *Evolution of extracellular DNA (eDNA) secretion in Bacillus subtilis.* Berlin: Springer.

[B14] Gomez-Brandon M. Ascher-Jenull J. Bardelli T. Fornasier F. Fravolini G. Arfaioli P. (2017a). Physico-chemical and microbiological evidence of exposure effects on Picea abies - Coarse woody debris at different stages of decay. *Forest Ecol. Manag.* 391 376–389. 10.1016/j.foreco.2017.02.033

[B15] Gomez-Brandon M. Ascher-Jenull J. Bardelli T. Fornasier F. Sartori G. Pietramellara G. (2017b). Ground cover and slope exposure effects on micro- and mesobiota in forest soils. *Ecol. Indic.* 80 174–185. 10.1016/j.ecolind.2017.05.032

[B16] Hamilton H. L. Domínguez N. M. Schwartz K. J. Hackett K. T. Dillard J. P. (2005). *Neisseria gonorrhoeae* secretes chromosomal DNA via a novel type IV secretion system. *Mol. Microbiol.* 55 1704–1721. 10.1111/j.1365-2958.2005.04521.x 15752195

[B17] Ibáñez de Aldecoa A. L. Zafra O. González-Pastor J. E. (2017). Mechanisms and regulation of extracellular DNA release and its biological roles in microbial communities. *Front. Microbiol.* 8:1390. 10.3389/fmicb.2017.01390 28798731 PMC5527159

[B18] Johnson B. J. Melde B. J. Dinderman M. A. Lin B. (2012). Stabilization of RNA through absorption by functionalized mesoporous silicate nanospheres. *PLoS One* 7:e50356. 10.1371/journal.pone.0050356 23226266 PMC3511576

[B19] Nagler M. Insam H. Pietramellara G. Ascher-Jenull J. (2018a). Extracellular DNA in natural environments: features, relevance and applications. *Appl. Microbiol. Biotechnol.* 102 6343–6356. 10.1007/s00253-018-9120-4 29858957 PMC6061472

[B20] Nagler M. Podmirseg S. M. Griffith G. W. Insam H. Ascher-Jenull J. (2018b). The use of extracellular DNA as a proxy for specific microbial activity. *Appl. Microbiol. Biotechnol.* 102 2885–2898. 10.1007/s00253-018-8786-y 29423636 PMC5847193

[B21] Pietramellara G. Ascher J. Borgogni F. Ceccherini M. T. Guerri G. Nannipieri P. (2009). Extracellular DNA in soil and sediment: fate and ecological relevance. *Biol. Fertil. Soils* 45 219–235. 10.1007/s00374-008-0345-8

[B22] R Core Team (2018). *R: A Language and Environment for Statistical Computing.* Vienna: R Foundation for Statistical Computing.

[B23] Steven B. Hesse C. Soghigian J. Gallegos-Graves V. Dunbar J. (2017). Simulated rRNA/DNA ratios show potential to misclassify active populations as dormant. *Appl. Environ. Microbiol.* 83:e00696-17. 10.1128/AEM.00696-17 PMC5440720 28363969

[B24] Wagner A. O. Malin C. Knapp B. A. Illmer P. (2008). Removal of free extracellular DNA from environmental samples by ethidium monoazide and propidium monoazide. *Appl. Environ. Microbiol.* 74 2537–2539. 10.1128/aem.02288-07 18296534 PMC2293149

