# Supplementary material for: Quantities of Intra- and Extracellular DNA Reveal Information About Activity and Physiological State of Methanogenic Archaea
Source: Front Microbiol. 2020 Aug 5;11:1894. doi: 10.3389/fmicb.2020.01894 (PMC7419480; doi:10.3389/fmicb.2020.01894)
Supplement: Supplementary file 1 [file Data_Sheet_1.docx]

Supplementary material

# Quantities of intra- and extracellular DNA reveal information about activity and physiological state of methanogenic archaea

Magdalena Nagler^1*^, Sabine Marie Podmirseg^1^, Markus Mayr^1^, Judith Ascher-Jenull^1^, Heribert Insam^1^

^1^Institute of Microbiology, Universität Innsbruck, Technikerstraße 25d, 6020 Innsbruck, Austria

**Correspondence:**

Magdalena Nagler

magdalena.nagler@uibk.ac.at

**Fig S1**


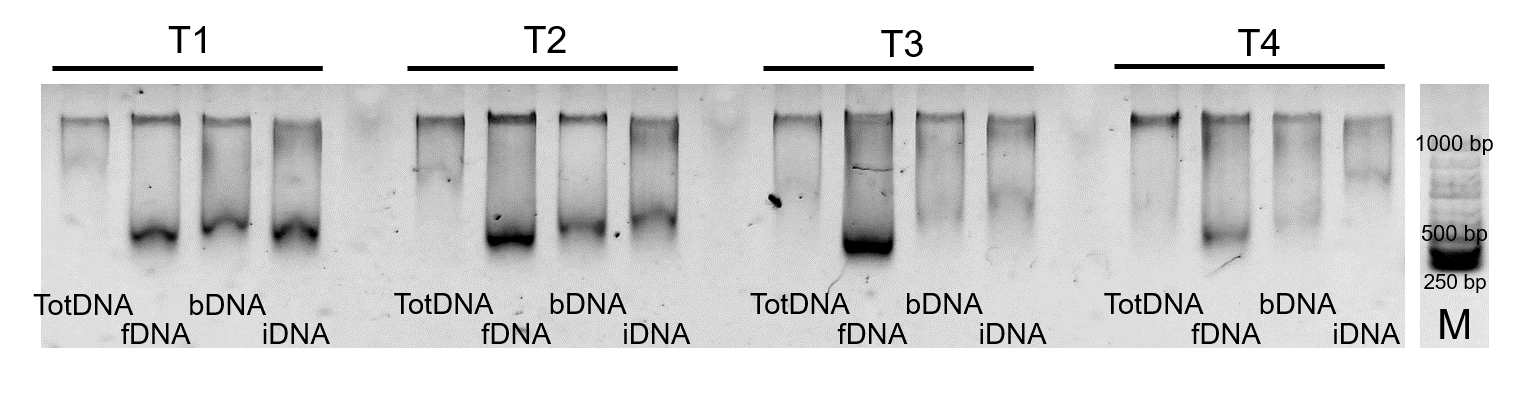


Agarose gel showing DNA samples of the total DNA pool (Tot; direct extraction) and all DNA fractions (sequential extraction; free extracellular DNA, fDNA; bound extracellular, bDNA; intracellular DNA, iDNA) of one sample belonging to the Rumen treatment from T1 to T4. Bands increase in sequence length from bottom to top ( M = Marker).

**Fig S2**

Comparison of mean methanogenic gene copy numbers in the f+b+iDNA and the totDNA, respectively. While HPP (high productive phase) samples show relatively high totDNA amounts with regard to f+b+iDNA, amounts of CL (cell lysis) samples are only marginally lower in f+b+iDNA as compared to totDNA. LPP (low productive phase).

**Fig S3**


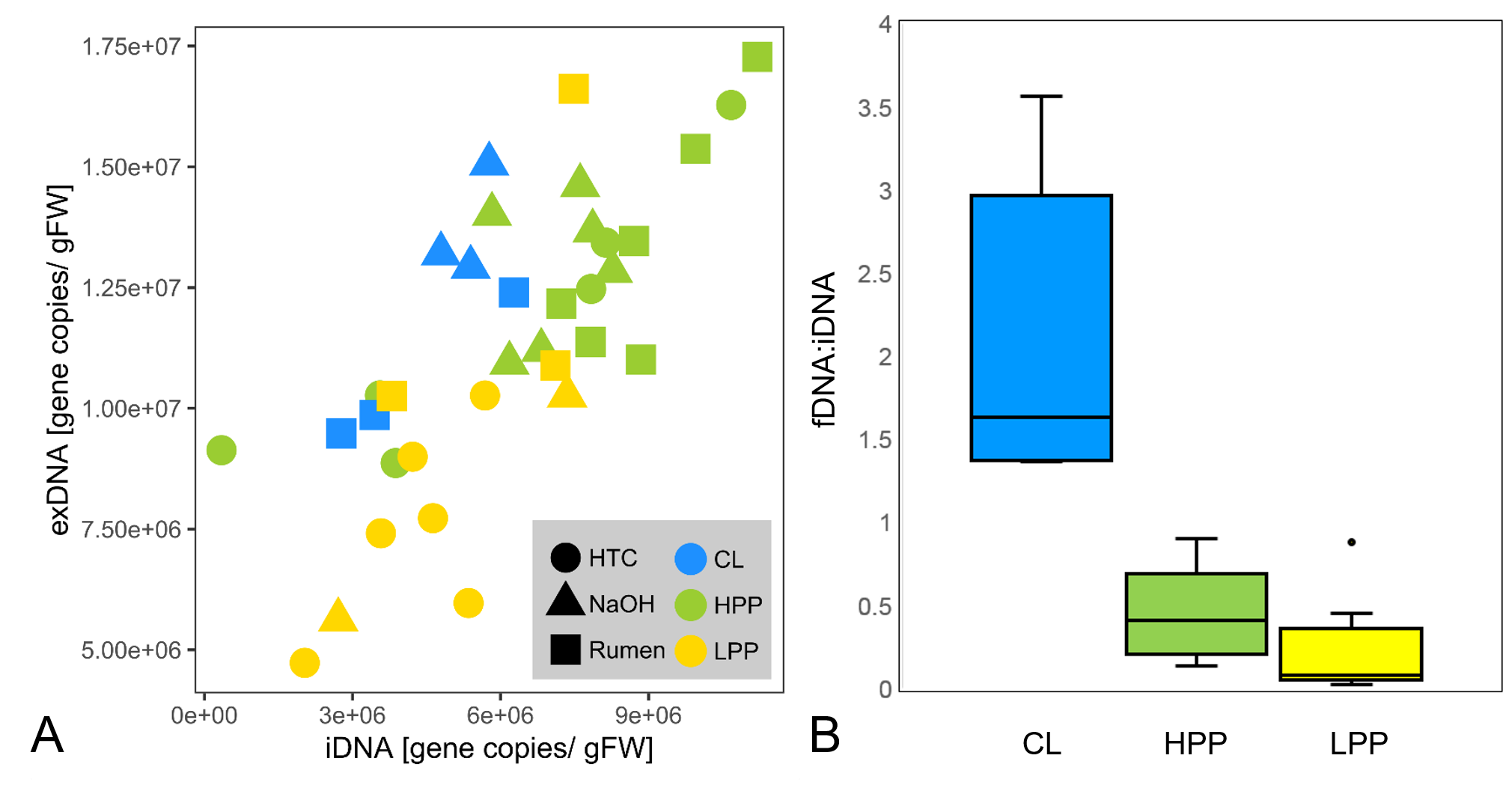


A) The relationship of exDNA and iDNA quantities and B) a boxplot of fDNA:iDNA-ratios of the physiological states of methanogenic archaea.

**Fig S4**


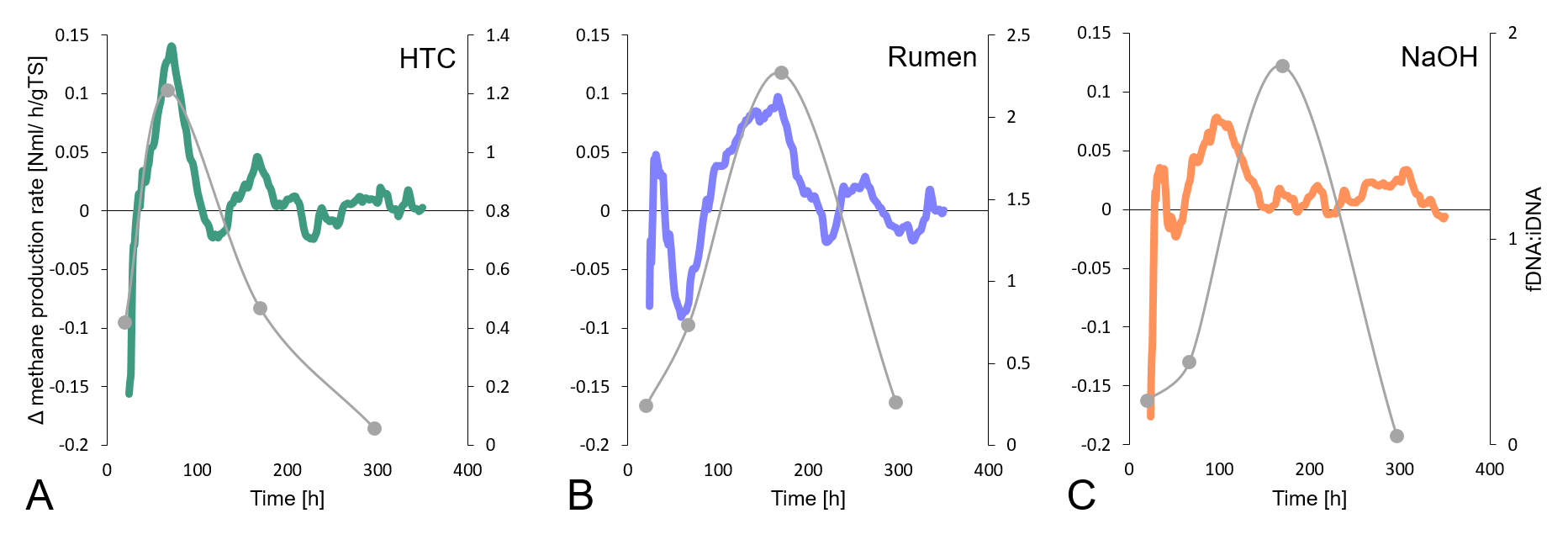


Inverse differences in methane prodcution rates (running mean of 20 hours) (colored lines) and corresponding fDNA:iDNA-ratios (grey lines). Peaks in colored lines depict a steep decrease in methane production rates, while peaks in fDNA:iDNA indicate high relative abundance of fDNA, presumably deriving from cell lysis.

**Fig. S5**


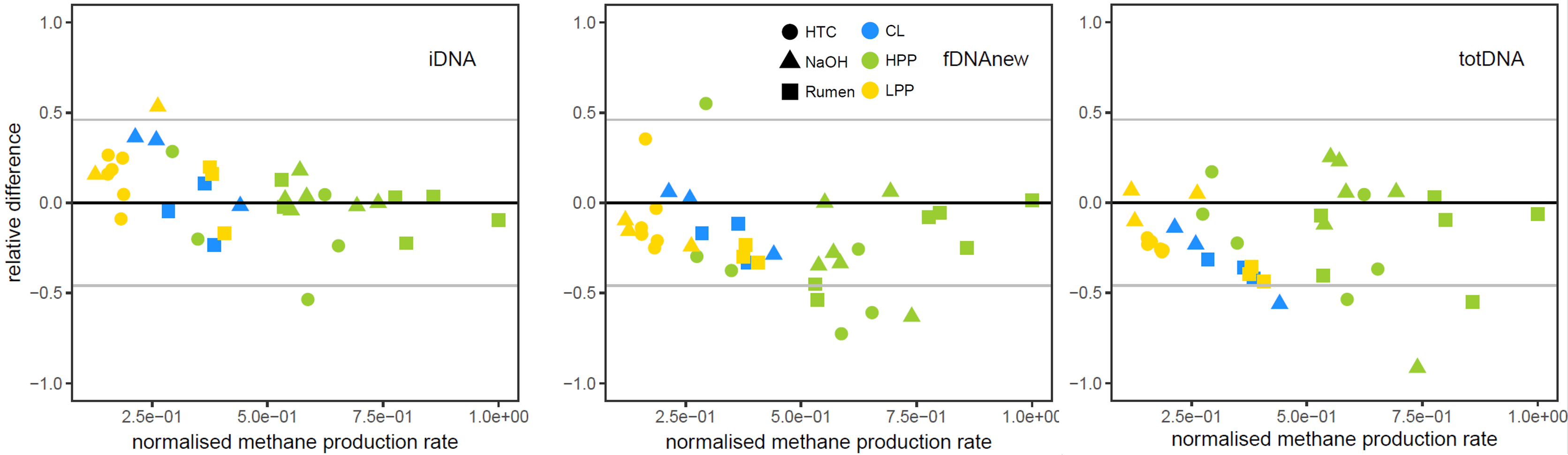


Deviations of normalized methanogenic gene copies of DNA fractions from the mean normalized methane production rate. Grey lines represent the two-fold standard deviation of the normalized methane production rate. Colors are based on the methanogenic activity levels (HPP= high productive phase, LPP= low productive phase, CL= cell lysis-influenced samples) while shapes correspond to treatments.
